# A Comprehensive Review of Biomarkers for Chronic Kidney Disease in Older Individuals: Current Perspectives and Future Directions

**DOI:** 10.7759/cureus.70262

**Published:** 2024-09-26

**Authors:** Aman Gupta, Tushar Sontakke, Sourya Acharya, Sunil Kumar

**Affiliations:** 1 Medicine, Jawaharlal Nehru Medical College, Datta Meghe Institute of Higher Education and Research, Wardha, IND

**Keywords:** aging, biomarkers, chronic kidney disease, cystatin c, early detection, kim-1

## Abstract

Chronic kidney disease (CKD) is a progressive condition characterized by a gradual loss of kidney function, leading to significant health complications and an increased risk of cardiovascular events. Early detection and effective management are crucial for slowing disease progression and improving patient outcomes. Biomarkers are valuable tools in CKD diagnosis, prognosis, and treatment. Traditional biomarkers, such as serum creatinine and urine protein, are widely used, but emerging biomarkers like cystatin C, kidney injury molecule-1 (KIM-1), and neutrophil gelatinase-associated lipocalin (NGAL) offer enhanced diagnostic precision and insights into disease severity. These advanced biomarkers are particularly important in older adults, who may present with age-related physiological changes and comorbid conditions that complicate CKD management. This review explores the current state of biomarker research in CKD, focusing on their application in older populations. It highlights the role of traditional and emerging biomarkers, discusses their relevance for early detection and prognosis, and examines future directions in biomarker research, including technological innovations and personalized medicine approaches. By integrating biomarkers into clinical practice, healthcare providers can achieve more accurate diagnoses, tailor treatments to individual patient needs, and potentially improve the overall management of CKD. Continued research and development in this field are essential for addressing the complexities of CKD and advancing patient care.

## Introduction and background

Chronic kidney disease (CKD) is a progressive and often asymptomatic condition characterized by a gradual decline in kidney function over time [1]. The kidneys, crucial for filtering waste products and excess fluids from the blood, maintaining electrolyte balance, and supporting metabolic homeostasis, become less effective as CKD progresses [2]. This deterioration can lead to a range of systemic complications, including increased cardiovascular risk, reduced quality of life, and ultimately, end-stage renal disease (ESRD), which requires dialysis or kidney transplantation. CKD is typically categorized into five stages based on the glomerular filtration rate (GFR) and kidney damage [3]. Stage 1 involves kidney damage with a normal or increased GFR (≥90 mL/min/1.73 m²), while Stage 2 is characterized by kidney damage with mildly decreased GFR (60-89 mL/min/1.73 m²). Stage 3 denotes a moderate decrease in GFR (30-59 mL/min/1.73 m²), Stage 4 indicates a severe decrease in GFR (15-29 mL/min/1.73 m²), and Stage 5 reflects kidney failure (GFR <15 mL/min/1.73 m²), often necessitating dialysis or transplantation [1]. Early detection and management of CKD are essential to slow disease progression, mitigate complications, and improve patient outcomes [4].

Biomarkers are measurable indicators that provide valuable insights into biological processes, pathogenic mechanisms, and responses to therapeutic interventions. In CKD, biomarkers are crucial for various aspects of patient care [5]. They aid in the early diagnosis of CKD, often detecting the disease before significant changes in traditional tests, like serum creatinine, become evident. This early identification allows for timely interventions that can slow disease progression. Additionally, biomarkers play a significant role in prognosis by offering insights into the severity of kidney damage and predicting adverse outcomes [6]. For example, biomarkers associated with inflammation or fibrosis can indicate the extent of kidney damage and the risk of progression to ESRD. Moreover, biomarkers are instrumental in treatment by helping to tailor therapies to individual patients and monitoring their responses to treatment [7].

This review aims to provide a comprehensive overview of the current state of biomarker research in CKD, with a particular focus on older individuals who may face unique challenges and considerations. It will explore traditional and emerging biomarkers, their relevance in aging populations, and potential future directions in this field. By enhancing our understanding of biomarkers in CKD, we aim to improve diagnosis, prognosis, and management strategies for this increasingly prevalent condition.

## Review

Methodology 

The search methodology for this comprehensive review on biomarkers for CKD in older individuals involves several structured steps to ensure a thorough and systematic approach. First, the research questions and objectives are clearly defined, focusing on identifying and evaluating both traditional and emerging biomarkers for CKD, their application in older adults, and potential future directions in this field. A comprehensive list of relevant keywords is developed, including primary terms such as CKD, biomarkers, and older adults, along with specific biomarkers like serum creatinine, cystatin C, kidney injury molecule-1 (KIM-1), and neutrophil gelatinase-associated lipocalin (NGAL). Synonyms and variations are also considered to broaden the search scope. Next, appropriate databases, such as PubMed, EMBASE, the Cochrane Library, and Google Scholar, are selected to cover a wide range of clinical, biomedical, and multidisciplinary literature. Search strategies are then formulated using a combination of Boolean operators to create effective search strings. For instance, a search string could be structured as (“Chronic Kidney Disease” OR CKD) AND (biomarker* OR “biological marker*”) AND (“older adults” OR elderly), ensuring that the search is both comprehensive and focused on the research questions. The inclusion and exclusion criteria are defined to guide the selection of studies. Inclusion criteria focus on studies involving biomarkers in CKD, with a particular emphasis on older adults and peer-reviewed articles, while exclusion criteria filter out studies not relevant to the topic, non-English language articles, and non-human studies. Following this, the search results are screened based on titles and abstracts, with studies meeting the inclusion criteria selected for full-text review. Data extraction is then conducted from the selected studies, focusing on aspects such as study design, population characteristics, biomarkers investigated, and key findings. These data are synthesized to compare the effectiveness and applicability of various biomarkers in diagnosing and managing CKD in older adults. A critical appraisal of the studies is performed using appropriate tools like CASP or PRISMA to assess their quality and reliability. To ensure the inclusion of the most recent and relevant studies, the search is updated shortly before finalizing the review. Throughout the process, detailed documentation of the search strategy, including databases searched, search strings used, and the number of results at each stage, is maintained for transparency and reproducibility. This structured methodology aims to provide a comprehensive and reliable synthesis of current research on CKD biomarkers in older adults.

Biomarkers in CKD

CKD is a major public health concern, particularly among older adults. The assessment and management of CKD heavily depend on biomarkers, which can be grouped into traditional, emerging, and those specific to certain aspects of the disease. Understanding these biomarkers is essential for improving early detection, monitoring disease progression, and tailoring treatment strategies [8]. Traditional biomarkers for CKD include serum creatinine, blood urea nitrogen (BUN), and urine protein and albumin. Serum creatinine is widely used to estimate kidney function and calculate the estimated GFR (eGFR). However, it has limitations, especially in detecting early kidney damage and in patients with preserved renal function [9]. BUN levels provide additional context for assessing kidney function but can be influenced by factors such as hydration status and protein intake, making them less reliable when used in isolation. Urine protein or albumin is a critical indicator of kidney damage, with albuminuria being particularly significant as it correlates with CKD progression and cardiovascular risk [10]. Emerging biomarkers are gaining prominence in nephrology, offering more sensitive indicators of kidney function and damage. Cystatin C is one such biomarker, increasingly recognized for its ability to detect early stages of CKD more accurately than serum creatinine, as it is less influenced by muscle mass and other confounding factors [11]. KIM-1 is another important urinary biomarker that indicates tubular injury and is associated with CKD progression and the need for renal replacement therapy [12]. NGAL is also noteworthy, reflecting both acute kidney injury and chronic kidney damage, and shows promise in predicting CKD progression and outcomes. Beta-2 microglobulin, linked to tubular function, has been associated with CKD progression and mortality, making it a valuable biomarker in clinical practice [12]. Certain aspects of CKD can be assessed through specific biomarkers. Inflammatory markers such as interleukin-6 (IL-6) and tumor necrosis factor-alpha (TNF-alpha) indicate systemic inflammation, which is often elevated in CKD patients and contributes to disease progression. Fibrosis markers, including transforming growth factor-beta 1 (TGF-beta 1) and various types of collagens, are involved in renal fibrosis, a common pathway in CKD progression [13]. Their levels can provide insights into the degree of kidney damage and the potential for recovery. Additionally, oxidative stress markers are crucial, as oxidative stress plays a significant role in kidney injury. Assessing these markers can help in understanding the underlying pathophysiology of CKD and guide therapeutic interventions [14]. Key biomarkers in CKD - traditional, emerging, and specific aspect indicators - are presented in Table 1.

**Table 1:** Key biomarkers in chronic kidney disease: traditional, emerging, and specific aspect indicators

Biomarker	Category	Description	Clinical relevance
Serum creatinine [15]	Traditional	A waste product from muscle metabolism measured in the blood.	Widely used to estimate the glomerular filtration rate (GFR) and assess kidney function.
Blood urea nitrogen (BUN) [16]	Traditional	Measures the amount of nitrogen in the blood that comes from urea.	It helps evaluate kidney function and hydration status, which diet and protein intake influence.
Urine protein and albumin [17]	Traditional	Detection of protein or albumin in urine samples.	An early indicator of kidney damage is used to monitor disease progression and treatment efficacy.
Cystatin C [18]	Emerging	A protein filtered by the kidneys serves as an alternative to creatinine for GFR estimation.	Provides a more accurate GFR estimation, especially in individuals with varying muscle mass.
KIM-1 (kidney injury molecule-1) [19]	Emerging	A protein expressed in injured kidney tubular cells.	Early detection of acute kidney injury and potential for monitoring chronic kidney disease (CKD) progression.
NGAL (neutrophil gelatinase-associated lipocalin) [20]	Emerging	A protein associated with kidney injury and inflammation.	Serves as an early marker for acute kidney injury and prognostic for CKD progression.
Beta-2 microglobulin [21]	Emerging	A low molecular weight protein cleared by the kidneys.	Indicates kidney function; elevated levels suggest renal dysfunction.
IL-6 (interleukin-6) [22]	Specific aspect (inflammation)	A pro-inflammatory cytokine is involved in immune responses.	Reflects inflammatory status in CKD; associated with cardiovascular risks and disease progression.
TNF-alpha (tumor necrosis factor-alpha) [23]	Specific aspect (inflammation)	A cytokine involved in systemic inflammation.	Linked to the progression of CKD and associated comorbid conditions.
TGF-beta 1 (transforming growth factor-beta 1) [24]	Specific aspect (fibrosis)	A cytokine involved in tissue fibrosis and scarring.	Marker of renal fibrosis associated with CKD progression and severity.
Collagen types (e.g., type IV) [25]	Specific aspect (fibrosis)	Structural proteins are involved in the formation of the extracellular matrix.	Indicators of fibrotic changes in kidney tissue are used to assess the extent of kidney damage.
Oxidative stress markers (e.g., malondialdehyde) [26]	Specific aspect (oxidative stress)	Molecules indicating oxidative damage and stress within the body.	Reflects oxidative stress status linked to CKD progression and associated complications.

Biomarker utilization in older adults

The utilization of biomarkers in older adults, especially for CKD, requires careful navigation due to the unique challenges posed by age-related physiological changes and comorbid conditions. These factors significantly influence the assessment and interpretation of biomarkers, making it essential for healthcare providers to grasp the nuances of biomarker data in this population [27]. One of the primary challenges in older adults is the impact of age-related physiological changes on biomarker levels. As individuals age, they undergo renal function, muscle mass, and body composition alterations, all of which can affect traditional biomarkers such as serum creatinine and albumin levels [28]. For example, the decline in muscle mass commonly seen in older adults may lead to lower serum creatinine levels, potentially masking underlying kidney dysfunction. This necessitates careful adjustments in the interpretation of biomarker data to prevent misdiagnosis or inappropriate management of CKD [28]. Moreover, the presence of comorbid conditions like diabetes, hypertension, and cardiovascular diseases further complicates the assessment of CKD biomarkers. These conditions can independently influence biomarker levels, introducing potential confounding factors in diagnosing and monitoring CKD in older adults. For instance, inflammation markers may be elevated due to comorbidities rather than kidney dysfunction, obscuring the clinical picture and complicating the determination of the appropriate course of treatment [7]. Given these challenges, it is crucial to consider age-specific adjustments when evaluating traditional biomarkers. The normal ranges for serum creatinine and eGFR may need to be recalibrated for older adults to account for decreased muscle mass and altered metabolism. Such recalibration ensures that clinicians can more accurately assess kidney function and detect early signs of CKD, ultimately leading to better management and outcomes for older patients [29]. The relevance of emerging biomarkers in older populations cannot be overstated. Biomarkers such as NGAL and KIM-1 hold promise for enhancing the detection and monitoring of CKD [30]. These emerging biomarkers may provide additional insights into kidney damage and function that are less influenced by age-related physiological changes. Their incorporation into clinical practice could improve early diagnosis and facilitate personalized treatment strategies for older adults with CKD [30]. The challenges, considerations, and clinical implications of biomarker utilization in older adults are outlined in Table 2.

**Table 2:** Biomarker utilization in older adults: challenges, considerations, and clinical implications

Aspect	Biomarker	Challenges in older adults	Considerations for clinical use
Traditional biomarkers [31]	Serum creatinine	Age-related decrease in muscle mass can lead to lower creatinine levels, underestimating kidney dysfunction.	Adjusting for muscle mass or using alternative biomarkers like cystatin C to better estimate kidney function.
	Blood urea nitrogen (BUN)	Age-related changes in protein intake and liver function may be affected, leading to variability in levels.	Monitor alongside creatinine and other kidney function markers for a more accurate assessment.
	Urine protein and albumin	Older adults may have higher baseline levels of proteinuria due to comorbid conditions (e.g., hypertension, diabetes).	Consider coexisting conditions; use thresholds adjusted for age when assessing kidney damage.
Emerging biomarkers [32]	Cystatin C	Cystatin C levels can better reflect kidney function in older adults and are less influenced by muscle mass than creatinine.	Increasingly used in older populations to improve glomerular filtration rate (GFR) estimation and assess chronic kidney disease (CKD) severity.
	KIM-1 (kidney injury molecule-1)	The exact impact of age on KIM-1 levels is still being researched, with the potential for variability due to comorbidities.	It is promising to detect early kidney injury, but age-related cutoffs may be necessary.
	NGAL (neutrophil gelatinase-associated lipocalin)	Chronic low-grade inflammation common in older adults may alter NGAL levels.	It could serve as an early marker for kidney injury, but further studies are needed on age-adjusted reference ranges.
Biomarkers for specific aspects of CKD [33]	Inflammatory markers (interleukin-6 (IL-6), tumor necrosis factor-alpha (TNF-alpha))	Levels may be elevated due to chronic inflammation associated with aging and comorbidities.	Essential for assessing systemic inflammation, but careful interpretation is needed to differentiate between CKD and aging.
	Fibrosis markers (transforming growth factor-beta1 (TGF-beta1), collagen types)	Age-related fibrosis and tissue remodeling can confound results, making distinguishing CKD-related fibrosis from aging effects difficult.	It is useful for assessing CKD progression but needs to account for age-related fibrotic changes.
	Oxidative stress markers	Oxidative stress levels increase with age, potentially confounding CKD-related findings.	It can indicate CKD progression, but age-specific baselines should be considered.
Impact of comorbid conditions [34]	All biomarkers	Comorbidities like diabetes, cardiovascular disease, and hypertension are common in older adults, influencing biomarker levels.	It is crucial to carefully evaluate comorbid conditions and their potential impact on CKD biomarkers.
Age-specific adjustments [35]	Serum creatinine, BUN, cystatin C	Physiological changes with aging (e.g., reduced GFR, decreased muscle mass) affect baseline biomarker levels.	Use age-adjusted equations for GFR (e.g., CKD-EPI (epidemiology) equation) and incorporate alternative biomarkers like cystatin C.
Clinical considerations in older adults [36]	All biomarkers	Age-related changes in renal physiology, polypharmacy, and frailty may affect biomarker reliability.	Multifactorial assessment is essential, considering overall health status and using multiple biomarkers for accurate diagnosis.

Current perspectives on biomarker research

Recent advancements in multi-omics, cytometry, and imaging, coupled with novel bioinformatics and biostatistics methodologies, have significantly accelerated the discovery and development of reproducible biomarkers for complex diseases. Among these, proteomics has emerged as a promising tool, enabling the discovery and validation of new protein markers through mass spectrometry [37]. A comparative study between older populations in the U.S. and China revealed that U.S. participants exhibited worse biomarker profiles and higher mortality rates associated with CKD, underscoring the need for tailored CKD management strategies that take demographic and lifestyle factors into account [38]. In cancer research, significant strides have been made in establishing reliable, cost-effective, and powerful diagnostic technologies. Pioneering studies are increasingly adopting a multidisciplinary approach to cancer diagnostics, moving beyond traditional screening methods to focus on various biomarkers, including nucleic acids, proteins, enzymes, and circulating tumor cells (CTCs) [39]. For CKD, the CKD-epidemiology (CKD-EPI) collaboration equation is currently preferred for its superior accuracy in estimating GFR, particularly at higher GFR levels. The combination of serum creatinine, cystatin C, and the urinary albumin-to-creatinine ratio has been shown to enhance risk stratification and improve predictions of CKD progression and mortality [40]. Albuminuria remains one of the most potent risk factors for CKD outcomes, being strongly associated with disease progression and cardiovascular risk. Emerging biomarkers such as NGAL, KIM-1, and liver-type fatty acid-binding protein (L-FABP) have been linked to kidney damage, disease severity, and progression [41]. In cancer diagnostics, recent trends include the development of novel multiplexed and integrated platforms that offer accurate and easy readouts, facilitating early cancer diagnosis and contributing to significant reductions in mortality. However, technical limitations and challenges in achieving high sensitivity and selectivity persist, underscoring the need for ongoing innovation in this field [42]. A comprehensive overview of current perspectives on biomarker research in CKD, including advances, challenges, and clinical implications, is provided in Table 3.

**Table 3:** Current perspectives on biomarker research in chronic kidney disease: advances, challenges, and clinical implications

Area of focus	Recent advances	Challenges	Clinical implications
Discovery of novel biomarkers [43]	Advances in omics technologies (genomics, proteomics, metabolomics) have identified new biomarker candidates.	Lack of standardization in biomarker discovery methods.	Potential to identify more specific and sensitive biomarkers for early chronic kidney disease (CKD) detection and prognosis.
Validation of biomarkers [44]	Increased focus on large-scale clinical trials to validate new biomarkers for CKD.	Difficulty in reproducing results across different populations and study designs.	Validation is crucial to ensure biomarkers can be reliably used in clinical practice for CKD diagnosis.
Biomarker panels [45]	Combining multiple biomarkers (e.g., panels including cystatin C, neutrophil gelatinase-associated lipocalin (NGAL), kidney injury molecule-1 (KIM-1)) to improve diagnostic accuracy.	Complexity in interpreting combined biomarker results, especially in populations with multiple comorbidities.	Biomarker panels may enhance the ability to detect CKD at earlier stages or differentiate between CKD causes.
Integration of artificial intelligence (AI) [46]	AI and machine learning are used to analyze complex biomarker datasets and improve predictive models.	Ensuring transparency and interpretability of AI-based models in clinical settings.	AI can provide more accurate predictions for CKD progression and personalize treatment plans based on biomarkers.
Personalized medicine [47]	Biomarker-driven personalized treatment approaches are being explored, allowing tailored interventions.	High cost and complexity of implementing personalized medicine in standard clinical practice.	Biomarkers can help customize therapies for individual patients, leading to more effective CKD management.
Non-invasive biomarker discovery [48]	Development of urine-based biomarkers (e.g., urinary NGAL, albumin) to provide non-invasive CKD monitoring.	Non-invasive biomarkers may be less sensitive or specific than blood-based biomarkers.	Non-invasive biomarkers offer a more patient-friendly approach to regular CKD monitoring and early detection.
Longitudinal biomarker monitoring [49]	Increasing emphasis on using biomarkers to monitor CKD progression over time in individual patients.	Biomarker levels can fluctuate due to factors unrelated to CKD, such as medications or infections.	Continuous monitoring of biomarkers can help adjust treatment strategies and assess disease progression.
Integration into clinical practice [50]	Development of guidelines for biomarker use in CKD diagnosis and management, such as the Kidney Disease: Improving Global Outcome (KDIGO) guidelines.	Lack of widespread adoption in clinical practice due to variability in biomarker availability and cost.	Guidelines help standardize the use of biomarkers in CKD management, improving diagnosis and treatment outcomes.
Biomarkers for specific CKD pathways [51]	Identifying biomarkers specific to inflammation, fibrosis, and oxidative stress in CKD.	Difficulty in distinguishing between CKD-related biomarker changes and those due to aging or comorbidities.	Biomarkers for specific pathways can help in targeted therapies for different stages and types of CKD.
Ethical and regulatory considerations [52]	Growing focus on ethical aspects, including patient consent and data privacy in biomarker research.	Regulatory hurdles for approving new biomarkers for clinical use.	Addressing ethical and regulatory issues is essential for safely and effectively implementing biomarkers.

Future directions

The future of biomarker research in CKD is poised to revolutionize diagnosis and treatment, with emerging innovations that could vastly enhance patient care. Novel biomarkers such as KIM-1, NGAL, and L-FABP are at the forefront of current investigations [53]. These biomarkers hold the potential to offer a more nuanced assessment of kidney health, enabling earlier intervention and improved patient outcomes. Their integration into clinical practice could enhance predictive models for CKD, resulting in more accurate risk stratification and tailored treatment plans [54]. The application of artificial intelligence (AI) and machine learning (ML) in biomarker research is rapidly gaining traction, particularly in analyzing complex datasets, identifying patterns, and refining risk stratification based on biomarker profiles [55]. AI algorithms can synergize clinical data with biomarker information to forecast disease progression and customize treatment strategies more effectively. This innovative integration paves the way for personalized medicine in CKD management, allowing for more individualized and effective care [55]. By leveraging individual biomarker profiles, healthcare providers can customize treatment plans that address the needs of older adults with CKD. This approach may involve adjusting medication regimens in response to biomarker feedback, implementing lifestyle modifications aligned with biomarker insights, and closely monitoring disease progression through targeted biomarker assessments [56]. Personalized medicine holds significant promise for older adults with CKD, who often contend with multiple comorbidities, as it can lead to more effective management strategies, reducing the risk of adverse outcomes and enhancing overall quality of life [56]. However, the path forward is not without challenges. The standardization and validation of novel biomarkers for clinical use remain critical hurdles. Ensuring these biomarkers are reliable, sensitive, and specific across diverse populations is essential for their successful incorporation into clinical practice. Continued research is needed to confirm the utility of these biomarkers in various clinical settings and to establish standardized protocols for their measurement [57]. Furthermore, the introduction of new biomarkers brings ethical considerations to the forefront, particularly concerning patient consent and the implications of biomarker testing. It is crucial that patients fully understand the purpose and potential outcomes of biomarker assessments to foster acceptance and trust. Addressing data privacy concerns and the use of AI in healthcare will also be vital as these technologies become more integral to CKD management [58].

## Conclusions

In conclusion, biomarkers play an essential role in managing CKD, offering critical insights into this progressive condition's diagnosis, prognosis, and treatment. As CKD continues to affect many individuals, particularly older adults who may present with unique clinical challenges, the advancement and application of biomarkers become increasingly important. Traditional biomarkers, such as serum creatinine and proteinuria, have been foundational in CKD management; however, emerging biomarkers provide new opportunities for early detection, more accurate risk assessment, and personalized treatment strategies. Understanding these biomarkers' roles and potential, particularly in the context of aging, can enhance clinical practice and patient outcomes. Future research and technological innovations will likely yield more precise biomarkers, improving our ability to manage CKD more effectively and tailor interventions to individual needs. This review underscores the importance of continued exploration and integration of biomarkers into CKD management to better address the disease's complexities and optimize patient care.
